# Computed Tomography Findings in Three Cases of Suspected Lymphovenous Communications in Cats

**DOI:** 10.3390/ani16203190

**Published:** 2026-10-11

**Authors:** Laura Librán-Ferreira, Iván Rodríguez-Armas, Carla Zamora-Perarnau, Nicolas Israeliantz, Vicente Cervera-Castellanos

**Affiliations:** 1 DIVET Teleradiology, 46003 Valencia, Spain; 2 Veterinary Referral Hospital UCV, 46018 Valencia, Spain; 3 Department of Small Animal Medicine and Surgery, Faculty of Veterinary Medicine and Experimental Sciences, Catholic University of Valencia San Vicente Mártir, 46001 Valencia, Spain; 4 Doctoral School, Catholic University of Valencia San Vicente Mártir, 46002 Valencia, Spain; 5 IDEXX Telemedicine Consultants, 70806 Kornwestheim, Germany

**Keywords:** lymphovenous communication, lymphatic system, thoracic duct, cisterna chyli, portal vein, computed tomography, cat, chylothorax

## Abstract

Pathological lymphovenous communications are poorly characterized in veterinary medicine, and their computed tomographic features and clinical significance remain unknown. This retrospective case series aimed to describe contrast-enhanced computed tomography findings of suspected spontaneous lymphovenous communications in three cats. All cats had dilated, tortuous vessels arising from portal venous tributaries and extending to the cisterna chyli, originating from the splenic vein in two cats and the cranial mesenteric vein in one. The cisterna chyli and thoracic duct were consistently dilated and contrast-enhancing, and reduced portal vein calibre cranial to the communications was present in all cats. Concurrent findings included pleural or peritoneal effusion and subcutaneous edema, with chylous pleural effusion confirmed in two cats. The recurrent anatomical distribution and associated portal and lymphatic abnormalities suggest that these communications may represent the enlargement and functional recruitment of pre-existing lymphovenous pathways in response to acquired portal and/or lymphatic hemodynamic disturbances, although a congenital origin cannot be excluded. Contrast-enhanced computed tomography may allow the recognition and anatomical characterization of suspected pathological lymphovenous communications in cats and should be considered when anomalous portal vessels are associated with concurrent cisterna chyli and thoracic duct abnormalities.

## Simple Summary

Lymphovenous communications are abnormal connections between the lymphatic and venous systems that are poorly characterized in veterinary medicine. This study describes the computed tomography findings of suspected lymphovenous communications in three older cats. In all cats, contrast-enhanced computed tomography identified abnormally dilated and tortuous vessels connecting branches of the portal venous system with the cisterna chyli, a major abdominal lymphatic structure. The cisterna chyli and thoracic duct were also dilated and showed contrast enhancement. Additional recurrent findings included narrowing of the portal vein, fluid accumulation within the thorax or abdomen, and subcutaneous edema. The similar anatomical distribution of the communications and the associated abnormalities suggest that these vessels may represent pre-existing connections that became enlarged and functionally relevant in response to chronic changes in portal or lymphatic circulation, although a congenital origin cannot be completely excluded. Recognition of this imaging pattern may help veterinarians identify previously unrecognized lymphovenous communications and improve the interpretation of complex portal and lymphatic abnormalities in cats undergoing contrast-enhanced computed tomography.

## 1. Introduction

The lymphatic system is formed by lymphatic vessels, trunks and ducts. The major lymphatic drainage pathways are organized around the thoracic duct (TD) and the right lymphatic duct (RLD) [1]. The TD represents the principal route for lymph return from most of the body, arising as the cranial continuation of the cisterna chyli (CC), terminating at the junction of the external and internal jugular veins or the junction between the jugular and left subclavian veins [1]. These terminal lymphovenous connections constitute the main physiological sites of communication between the lymphatic and venous systems. Within the abdomen, lymph from the viscera reaches the CC through several lymphatic trunks, including the intestinal and celiac trunks [1]. Anatomical studies have demonstrated considerable variability in the organization of these lymphatic pathways, particularly the intestinal trunk, whose tributaries may form an extensive interconnected lymphatic network (rete intestinale) surrounding the cranial mesenteric artery before entering the cisterna chyli [2].

In human medicine, the lymphovenous communications (LVCs) classified as normal and commonly seen are the thoracic duct termination, right lymphatic duct termination and intra-nodal LVCs [3]. Pathological LVCs have also been described in a variety of disease states, including primary and secondary lymphedema, as well as primary and metastatic malignancies involving the lymph nodes [3]. In addition, lymphovenous communications can be performed surgically for the management of secondary lymphedema and other lymphatic disorders [3].

Regarding veterinary medicine, Threefoot (1968) [4] demonstrated physiologic LVCs in several mammalian species, including dogs, involving multiple venous territories throughout the body, while Zajac (1972) [5] described communications between the cisterna chyli and the caudal vena cava. Furthermore, experimental studies by Griaznova (1962) [6] and by Kagan and Breznock (1979) [7] showed that complete thoracic duct obstruction resulted in the opening of LVCs proximal to the site of obstruction, supporting their role as compensatory pathways for maintaining lymphatic drainage. Acquired LVCs are also believed to play an important role in the success of the TD ligation procedures for the treatment of chylothorax [8]. More recently, experimental studies in dogs and cats have demonstrated the technical feasibility of creating direct LVCs between the thoracic duct and the venous system as a potential therapeutic strategy for lymphatic disorders [9,10].

However, pathological LVCs remain poorly characterized in veterinary medicine, with the available literature largely limited to anatomical observations and experimental studies. Consequently, their clinical significance, imaging characteristics, and potential association with underlying disease remain largely unknown. To the authors’ knowledge, this is the first case series describing suspected spontaneous LVCs in cats using contrast-enhanced computed tomography, providing novel information on their anatomical distribution, imaging features, and associated abnormalities.

## 2. Materials and Methods

In this retrospective study, cases of presumptive lymphovenous communications were identified from teleradiology submissions between 2024 and 2025 using the search terms “lymphovenous shunt” and “lymphaticovenous communication.” Cats were included if a complete contrast-enhanced CT examination of the thorax and abdomen was available and demonstrated the following: (1) an anomalously dilated and tortuous vascular structure arising from or showing direct continuity with a tributary of the portal venous system; (2) a traceable anatomical course connecting this structure with the CC and/or TD. CT studies were required to be of sufficient diagnostic quality to evaluate the portal venous and lymphatic structures involved. Four cats with suspected LVCs were initially identified. Three fulfilled the inclusion criteria and were included in the study, whereas one cat was excluded because only a thoracic CT examination was available, precluding assessment of the abdominal origin and complete course of the suspected communication. No suspected LVCs involving other anatomical locations were identified during the database search. Referring veterinarians were contacted and requested to provide all available clinical information, including signalment, clinical history, previous diagnostic investigations and treatment, physical examination, blood analysis, cytology/histopathology, and clinical outcome.

Different CT machines and protocols were used depending on the referring institution: Cases 1 and 2 were imaged using a Aquilion Start 16-detector-row CT scanner (Canon Medical Systems, Otawara, Japan). Image acquisition was performed in helical mode using a slice thickness of 1.0 mm, pitch of 0.94, gantry rotation time of 0.75 s, tube voltage of 100 kVp, and tube current of 90 mA. Case 3 was examined using a SOMATOM 32-detector-row CT scanner (Siemens Healthineers, Erlangen, Germany) with a slice thickness of 2 mm, pitch of 1.2, gantry rotation time of 0.33 s, tube voltage of 100 kVp, and tube current of 41 mA.

All protocols were performed in ventral recumbency under general anaesthesia. They included soft tissue and bone algorithm images of the entire abdomen, before and after intravenous iodinated contrast medium administration (Iohexol, Omnipaque^®^, GE Healthcare, Oslo, Norway), at 600 mg/kg with unspecified phase protocols.

Each CT study was carefully reviewed by two board-certified veterinary radiologists (V.C.C. and N.I.), one second-year diagnostic imaging resident (L.L.F.) and one diagnostic imaging intern (I.R.A.) using an image analysis workstation (Horos, version 3.3.6, Horos Project, Annapolis, MD, USA). The following characteristics were reviewed and recorded: the identification of an anomalous lymphovenous connection with associated lymphatic ducts via contrast enhancement; origin, path, drainage and size of the communication; the presence of peritoneal or pleural effusion; and structural conditions such as masses, lymph node affection or chronic inflammatory changes. All assessments were reached by consensus among the reviewers, who were aware of patient’s outcomes at the time of interpretation.

## 3. Results

### 3.1. Signalment, Clinical Signs, Physical Examination and Blood Laboratory Findings

A total of three cats were included. Signalment and clinical signs are summarized in Table 1.

CT examination was performed to investigate pleural effusion and respiratory distress in cases 1 and 3, with concurrent abdominal effusion in case 1. In case 2, CT was performed as part of the diagnostic investigation of the neurological signs and increased pre- and postprandial bile acid concentrations, which raised suspicion of hepatobiliary dysfunction.

### 3.2. Computed Tomography Findings

In case 1, contrast-enhanced CT revealed an abnormally dilated and tortuous vessel arising from the splenic vein and coursing craniodorsally toward the CC (Figure 1). The anomalous vessel entered the right lateral aspect of the CC at approximately the level of the intervertebral disc space between the second and third lumbar vertebrae (L2 and L3). The CC and TD were dilated and showed marked homogeneous enhancement following intravenous contrast administration. Cranially, the TD extended through the dorsal mediastinum, dividing into multiple interconnected branches before terminating within the systemic venous circulation at the level of the left external jugular vein, at its anastomosis with the left brachiocephalic vein. A mild reduction in portal vein calibre was identified cranial to the communication.

An irregular, strongly contrast-enhancing mass centered on the ileocecal region and cranial mesenteric lymphocenter was observed. The mass was associated with regional lymphadenopathy and encased the cranial mesenteric vessels and portal tributaries without evidence of overt vascular invasion. Bilateral pleural effusion with secondary pulmonary atelectasis and moderate to severe peritoneal effusion were also present. Additional findings included subcutaneous fluid accumulation along the ventral abdominal wall, collateral vascularization inserting in the left renal vein, and a small hypoattenuating hepatic nodule.

Case 2 (Figure 2) showed a network of tortuous, dilated vessels that originated from the cranial mesenteric vein and coursed dorsomedially, medial to the right kidney and caudal vena cava, before reaching the CC. A double origin was suspected, together with two separate insertions into the right lateral aspect of the CC at approximately the level of L2 and L3, one slightly cranial to the other. The CC and TD were moderately distended and demonstrated homogeneous contrast enhancement equal in attenuation to the portal venous system. The TD extended cranially between the aorta and azygos vein, bifurcating at approximately eleventh thoracic vertebrae (T11), with further division of the larger branch at the level of ninth thoracic vertebrae (T9). The portal vein was mildly reduced in calibre cranial to the communication.

Other abdominal findings consisted of minimal peritoneal effusion, mild subcutaneous fluid accumulation along the caudoventral abdominal wall, and mild multifocal intra- and extrahepatic biliary dilatation. The pancreatic duct was also mildly and diffusely dilated. A poorly defined, poor contrast-enhancing hepatic nodule was present within the quadrate hepatic lobe.

In case 3, an anomalous communication originated from a moderately dilated splenic vein and followed a tortuous craniodorsal course toward the CC (Figure 3). The main insertion was identified on the right lateral aspect of the CC around the level of L2 and L3, with additional insertions involving the left lateral aspect at the L3 and the right lateral aspect at fourth lumbar vertebra (L4). The CC and TD were distended and diffusely contrast-enhancing. The TD coursed cranially through the dorsal mediastinum and divided into multiple branches before draining into the systemic venous circulation. The portal vein was moderately decreased in calibre cranial to the communication.

Additional findings included marked dilatation and tortuosity of the left colic vein, with heterogeneous increased attenuation of the adjacent peritoneal fat. Bilateral pleural effusion with secondary pulmonary atelectasis and subcutaneous fluid accumulation at the thoracic inlet were also present. Multifocal, well-defined hypoattenuating hepatic nodules with poor contrast enhancement were also identified.

### 3.3. Ancillary Diagnosis, Clinical Management, Cytological/Histopathological Findings and Outcome

In case 1, cytological analysis of the pleural and abdominal effusions was consistent with a non-septic inflammatory process with abundant mature lymphocytes. Biochemical analysis of the pleural effusion confirmed its chylous nature, with a triglyceride concentration > 375 mg/dL compared with 165 mg/dL in serum. Cytology of the abdominal mass and regional lymph nodes revealed an undifferentiated neoplasm with marked cellular atypia, considered most consistent with a sarcomatous process. Echocardiography showed increased echogenicity of the parietal pericardium without pericardial effusion or evidence of constrictive disease. Progressive clinical deterioration associated with recurrent pleural and abdominal effusions and suspected neoplastic disease ultimately led to euthanasia.

In case 2, no further invasive diagnostic procedures were pursued following CT examination at the owners’ request, and the long-term outcome was unknown.

In case 3, cytological analysis of the pleural effusion was consistent with a chylous effusion, and a SNAP Feline proBNP test was negative. The cat was subsequently euthanized, and post-mortem tissue sampling was not performed.

No surgical exploration or treatment of the suspected lymphovenous communications was performed in any of the three cats, and histopathological confirmation of the communications was unavailable in all cases.

## 4. Discussion

The present cases provide the first tomographic characterization of suspected lymphovenous communications involving the portal venous and lymphatic systems in feline patients and represent, to the authors’ knowledge, the first radiological description of this phenomenon in veterinary medicine.

The main CT features shared by the three cats were dilated and tortuous vessels extending from portal venous tributaries to the CC, associated with dilatation and contrast enhancement of the CC and TD. Despite variation in their portal venous origin and number of lymphatic insertions, the communications followed a similar anatomical course. Additional recurrent findings included reduced portal vein caliber cranial to the communication and variable combinations of pleural or peritoneal effusion and subcutaneous edema. The consistent association of portal and lymphatic abnormalities across cases may reflect a common underlying hemodynamic disturbance and may raise suspicion for abnormal LVCs in cats undergoing contrast-enhanced CT.

The nature and origin of the suspected LVCs identified in these cats remain uncertain, and two main hypotheses may be considered. First, the anomalous vessels may represent true lymphovenous shunts permitting the passage of contrast-enhanced blood into the lymphatic circulation, as pathological lymphovenous shunting has been described in association with lymphatic disease in human patients [3,11]. Alternatively, these vessels may represent pre-existing and normally inconspicuous lymphovenous connections located within the extensive intestinal lymphatic network that become recruited, dilated, and functionally significant in response to acquired hemodynamic disturbances [2]. The recurrent anatomical location of these communications may also be explained by the normal organization of the abdominal lymphatic system. Baum (2023) [2] described an extensive intestinal lymphatic network receiving drainage from the jejunal, colic, splenic, hepatic, and lumbar territories before entering the CC, with considerable variability in the number and arrangement of its lymphatic channels [2]. This anatomical organization is particularly relevant to the present cases, as the communications consistently involved the splenic or cranial mesenteric veins and entered the CC at similar lumbar levels. Previous anatomical and experimental veterinary studies have also demonstrated spontaneous LVCs and recruitment of lymphovenous pathways following thoracic duct obstruction [4,5,6,7].

Increased portal pressure is known to increase hepatic lymph production and TD lymph flow [12,13], whereas increased lymphatic pressure or impaired lymphatic drainage may promote recruitment and enlargement of pre-existing lymphovenous communications [3,6,7,11,14]. The consistent dilatation of the CC and TD in the present cases, together with concurrent abnormalities potentially affecting portal or lymphatic drainage, may be compatible with the recruitment and enlargement of pre-existing lymphovenous pathways. In case 1, the infiltrative ileocecal and cranial mesenteric mass, regional lymphadenopathy, and encasement of mesenteric and portal tributaries could have impaired both venous and lymphatic drainage, whereas cases 2 and 3 showed hepatobiliary and portal vascular abnormalities, respectively, without an overt abdominal mass. In case 2, increased pre- and postprandial bile acid concentrations further supported hepatobiliary dysfunction, although their relationship with the vascular abnormalities identified on CT could not be established.

The recurrent effusions and subcutaneous edema may similarly reflect altered lymphatic hemodynamics rather than being a direct consequence of the communications. Increased lymph production and/or impaired TD drainage could exceed the transport capacity of the lymphatic system, resulting in lymphatic distension and increased hydrostatic pressure ultimately promoting leakage of chyle into body cavities [13,15,16]. In our cases, TD dilatation and subcutaneous edema was present in all three cats, while two had pleural effusion compatible with chylothorax and two had abdominal effusion compatible with chyloabdomen. However, neither lymphatic hypertension nor a causal relationship between these findings and the suspected LVCs could be demonstrated.

The advanced age of all three cats and the absence of previously recognized clinical manifestations may be more consistent with communications that became functionally relevant later in life. Nevertheless, previous cross-sectional imaging was unavailable, and a congenital origin cannot be excluded, particularly in case 2 because of its more complex configuration and multiple insertions. Overall, the combination of radiological findings raises the possibility that pre-existing lymphovenous pathways became recruited and dilated in response to altered portal and/or lymphatic hemodynamics; however, this should be regarded as a proposed mechanism rather than a demonstrated pathogenesis.

The direction of flow through these communications is particularly relevant to their interpretation. The reduction in calibre of the portal vein cranial to these communications, together with the marked enhancement of the anomalous vessels following intravenous contrast administration and the distension of the CC and TD cranial to the insertion of these communications, supports portal-to-lymphatic flow at the time of image acquisition. Although direct pressure measurements were not available, and portal hypertension therefore remains suspected rather than confirmed. Increased portal pressure could provide the pressure gradient required for contrast-enhanced portal blood to enter the lymphatic system through these communications.

Acquired portal venous collateralization secondary to portal hypertension was therefore an important differential consideration. However, conventional portal collaterals would be expected to provide alternative drainage from the portal circulation into the systemic venous system, whereas the anomalous vessels in all three cats consistently terminated within the CC and were associated with TD dilatation and contrast enhancement. This recurrent lymphatic involvement supports the presumptive interpretation of these vessels as lymphovenous communications rather than conventional acquired portal collaterals.

Contrast-enhanced CT was essential for recognizing the communications because it allowed simultaneous evaluation of the portal tributaries, anomalous vessels, CC, and TD. Enhancement of the feline lymphatic system following routine intravenous contrast administration has been reported under physiological conditions, particularly on delayed acquisitions [17,18]. However, the additional findings of direct anatomical continuity between portal tributaries and lymphatic structures, together with the avid lymphatic enhancement and dilatation cranial to the site of communication, were considered suggestive of pathological LVCs rather than normal lymphatic enhancement.

Based on a thorough review of the existing literature, this case series represents the first description of suspected natural occurring, pathological LVCs in feline patients using contrast-enhanced CT. While physiological and pathological LVCs have been previously described in human medicine in association with lymphatic disorders, portal hypertension, neoplasia, and lymphatic obstruction [3,12], the veterinary literature remains limited to anatomical observations [4,5], experimental models of TD obstruction [6,7], and surgically created lymphovenous anastomoses [9,10].

This study has several limitations, including the small number of cases, retrospective design, incomplete long-term follow-up, and the absence of surgical or histopathological confirmation of the suspected LVCs. The retrospective nature of the study also resulted in heterogeneous clinical and diagnostic data availability among patients and a lack of standardized diagnostic and imaging protocols across cases. Most importantly, neither portal nor lymphatic pressures were measured, and the direction of flow could not be directly demonstrated. Furthermore, lymphography was not performed in any of the cases, including cases 1 and 3, in which chylous pleural effusion was identified. As this was a retrospective multicentre case series based on cases submitted for teleradiology interpretation, diagnostic work-up was determined independently by the referring institutions, and lymphographic studies were not performed at the time of clinical investigation. This represents an important limitation, as lymphographic evaluation could have provided additional information regarding central lymphatic anatomy and drainage and allowed further characterization of the suspected communications, including contrast transit and flow direction. Although the concurrent enhancement of venous and lymphatic structures supported direct communication between both systems, the absence of lymphography precluded the definitive characterization of these vessels and their relationship with the central lymphatic system. An additional limitation is that the exact post-contrast acquisition phases were unavailable for all studies, preventing precise assessment of the temporal relationship between vascular and lymphatic enhancement.

## 5. Conclusions

In conclusion, this case series describes the CT features of suspected acquired, pathological LVCs in three cats. The findings described in this case series suggest that lymphovenous communications involving the portal venous system and lymphatic system may represent manifestations of adaptative lymphatic-venous remodelling in cats. Based on their similar anatomical distribution and the known organization of the feline intestinal lymphatic network, these communications may arise though recruitment and dilation of pre-existing lymphovenous pathways rather than de novo vascular formation in response to chronic disturbances in portal or lymphatic hemodynamics.

## Figures and Tables

**Figure 1:**
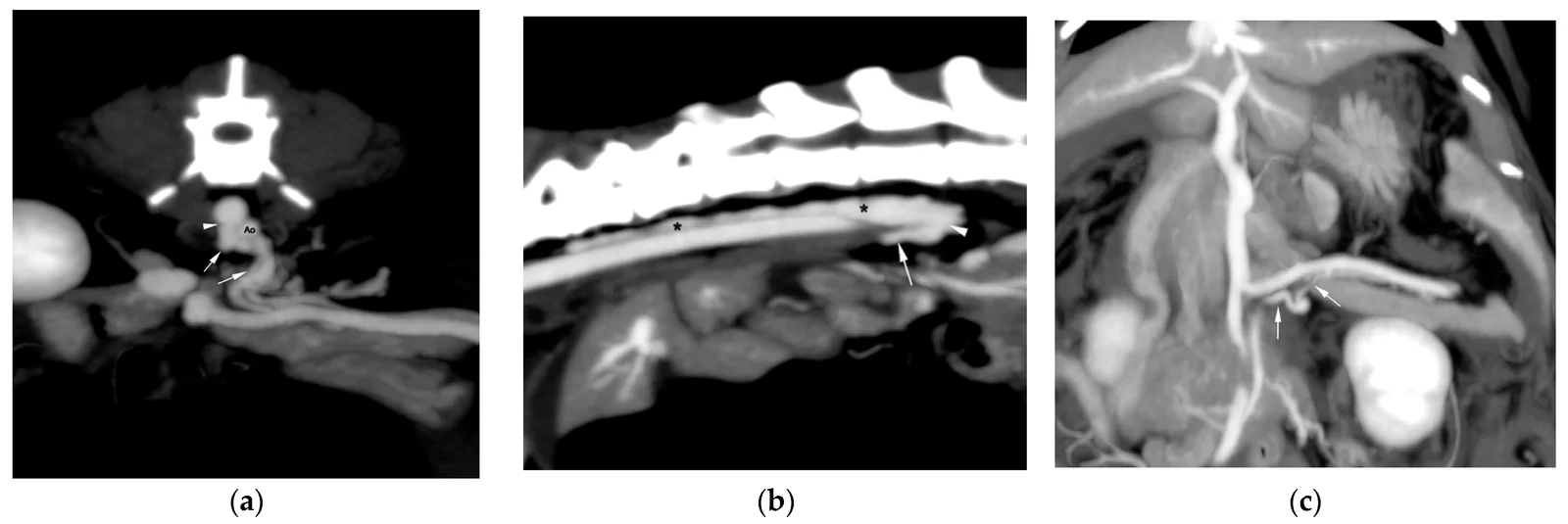
Maximum intensity projection postcontrast CT images from case 1. (**a**) Transverse multiplanar reconstruction of the distal aspect of the anomalous vessel (arrows) and its insertion in the cisterna chyli (arrowhead), to the right of the aorta artery (Ao). (**b**) Sagittal multiplanar reconstruction of the insertion of the anomalous vessel (arrow) into the cisterna chyli (arrowheads) with associated distension of the thoracic duct (asterisks). (**c**) Dorsal multiplanar reconstruction of the origin of the anomalous vessel (arrows) in the splenic vein.

**Figure 2:**
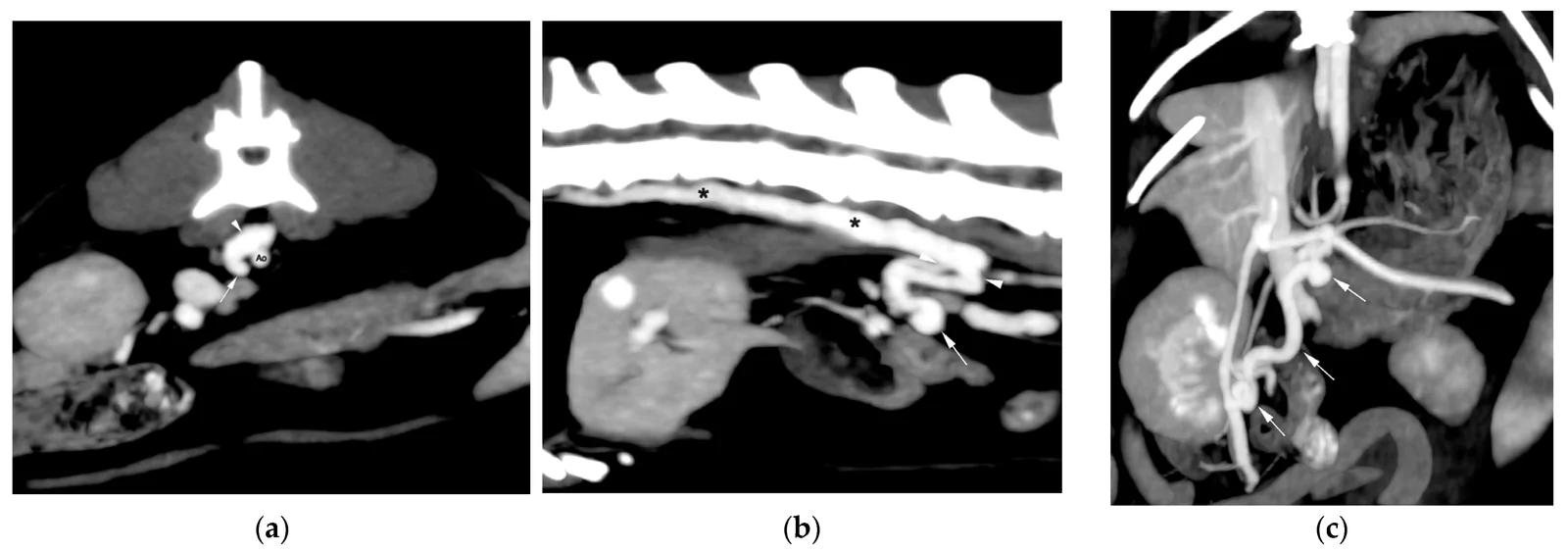
Maximum intensity projection postcontrast CT images from case 2. (**a**) Transverse multiplanar reconstruction of the distal aspect of the anomalous vessel (arrow) and its cranial insertion in the cisterna chyli (arrowhead) to the right of the aorta artery (Ao). (**b**) Sagittal multiplanar reconstruction of the two insertions of the anomalous vessel (arrow) into the cisterna chyli (arrowheads) with associated distension of the thoracic duct (asterisks). (**c**) Dorsal multiplanar reconstruction of the anomalous and tortuous vessel (arrows) arising from the cranial mesenteric vein.

**Figure 3:**
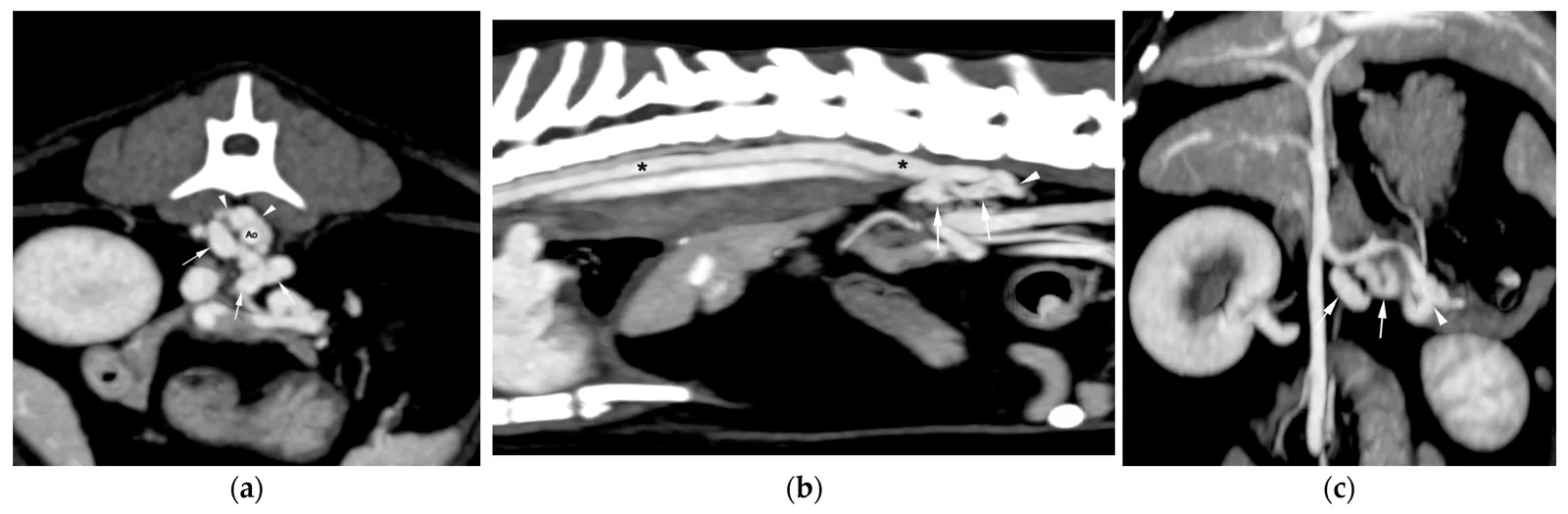
Maximum intensity projection postcontrast CT images from case 3. (**a**) Transverse multiplanar reconstruction of the distal aspect of the anomalous vessel (arrows) and its cranial insertions in the cisterna chyli (arrowheads) to the right and left of the aorta artery (Ao). (**b**) Sagittal multiplanar reconstruction of the caudal insertion of the anomalous vessel (arrows) in the cisterna chyli at the level of L4 (arrowheads) with associated secondary distension of the thoracic duct (asterisks). (**c**) Dorsal multiplanar reconstruction of the anomalous and tortuous vessel (arrows) arising from the splenic vein (arrowhead).

**Table 1:** Signalment, clinical signs, physical examination and laboratorial findings.

Case	Signalment	Clinical Signs and Physical Examination	Laboratorial Findings
1	10-year-old neutered male Domestic shorthair 5.2 kg	Respiratory distress associated with pleural and abdominal effusion. Mild dehydration (3%).	Moderate non-regenerative anemia (HCT 22.6%; HGB 6.9 g/dL). Thrombocytopenia (116 K/µL). Marked reticulocytosis (231.2 K/µL). Increased feline pancreatic lipase (fPL 15.4 ng/mL). Mild hyperglycemia and hypochloremia.
2	14-year-old neutered male Siamese 3.9 kg	Post-ictal stupor. Severe tachycardia (230 bpm), tachypnea (60 rpm), pale mucous membranes, hypothermia (36 °C), dehydration, and low body condition score (3/9).	Moderate non-regenerative anemia (HCT 19.2%; HGB 6.9 g/dL). Marked neutrophilic leukocytosis. Lymphopenia and eosinopenia compatible with stress leukogram. Mild hyperglycemia and azotemia. Markedly increased pre- and post-prandial bile acids (21.2 and 45.2 µmol/L).
3	16-year-old neutered male Siamese 4.2 kg	Acute respiratory distress with restrictive respiratory pattern associated with pleural effusion, grey and dry mucous membranes.	Mild leukocytosis (25.04 K/µL) due to moderate neutrophilia (22.76 K/µL) and subtle monocytosis (0.7 K/µL). Mild hypokalemia (3.4 mmol/L) and mild decrease in total calcium (7.9 mg/dL).

Abbreviations: HCT, hematocrit; HGB, hemoglobin; fPL, feline pancreatic lipase; bpm, beats per minute; rpm, respirations per minute.

## Data Availability

The data presented in this study are not publicly available due to privacy protection.

## Abbreviations

The following abbreviations are used in this manuscript:

Ao	Aorta artery
CC	Cisterna chyli
CT	Computed tomography
fPL	Feline pancreatic lipase
HCT	Hematocrit
HGB	Hemoglobin
L2	Second lumbar vertebrae
L3	Third lumbar vertebrae
L4	Fourth lumbar vertebrae
LVCs	Lymphovenous communications
proBNP	pro-B-type natriuretic peptide
RLD	Right lymphatic duct
T9	Ninth thoracic vertebrae
T11	Eleventh thoracic vertebrae
TD	Thoracic duct
